# Exploring the factors contributing to low vaccination uptake for nationally recommended routine childhood and adolescent vaccines in Kenya

**DOI:** 10.1186/s12889-023-15855-w

**Published:** 2023-05-19

**Authors:** Tene-Alima Essoh, Gbadebo Collins Adeyanju, Abdu A. Adamu, Haoua Tall, Aristide Aplogan, Collins Tabu

**Affiliations:** 1 Agence de Médecine Préventive (AMP) Afrique, Cote d’Ivoire, Abidjan, Côte d’Ivoire; 2 Center for Empirical Research in Economics and Behavioral Science (CEREB), University of Erfurt, Erfurt, Germany; 3 Psychology and Infectious Disease Lab (PIDI), Media and Communication Science, University of Erfurt, Erfurt, Germany; 4 Bernhard Nocht Institute of Tropical Medicine (BNITM), Hamburg, Germany; 5 South African Medical Research Council, Cochrane South Africa, Cape Town, South Africa; 6 Division of Epidemiology and Biostatistics, Department of Global Health, Faculty of Medicine and Health Sciences, Stellenbosch University, Cape Town, South Africa; 7 Kenya Medical Research Institute (KEMRI), Welcome trust, Nairobi, Kenya; 8 National Vaccines and Immunization Program, Ministry of Health, Nairobi, Kenya

**Keywords:** Vaccine hesitancy, Vaccine demand, Low uptake, HPV vaccine, Human papillomavirus, Immunization, Vaccination, Adolescent girls, Infectious diseases, Girls, Women

## Abstract

**Background:**

Vaccination remains the most effective means of reducing the burden of infectious disease among children. It is estimated to prevent between two to three million child deaths annually. However, despite being a successful intervention, basic vaccination coverage remains below the target. About 20 million infants are either under or not fully vaccinated, most of whom are in Sub-Saharan Africa region. In Kenya, the coverage is even lower at 83% than the global average of 86%. The objective of this study is to explore the factors that contribute to low demand or vaccine hesitancy for childhood and adolescent vaccines in Kenya.

**Methods:**

The study used qualitative research design. Key Informant Interviews (KII) was used to obtain information from national and county-level key stakeholders. In-depth Interviews (IDI) was done to collect opinions of caregivers of children 0–23 months and adolescent girls eligible for immunization, and Human papillomavirus (HPV) vaccine respectively. The data was collected at the national level and counties such as Kilifi, Turkana, Nairobi and Kitui. The data was analyzed using thematic content approach. A total of 41 national and county-level immunization officials and caregivers formed the sample.

**Results:**

Insufficient knowledge about vaccines, vaccine supply issues, frequent healthcare worker’s industrial action, poverty, religious beliefs, inadequate vaccination campaigns, distance to vaccination centers, were identified as factors driving low demand or vaccine hesitancy against routine childhood immunization. While factors driving low uptake of the newly introduced HPV vaccine were reported to include misinformation about the vaccine, rumors that the vaccine is a form of female contraception, the suspicion that the vaccine is free and available only to girls, poor knowledge of cervical cancer and benefits of HPV vaccine.

**Conclusions:**

Rural community sensitization on both routine childhood immunization and HPV vaccine should be key activities post COVID-19 pandemic. Likewise, the use of mainstream and social media outreaches, and vaccine champions could help reduce vaccine hesitancy. The findings are invaluable for informing design of context-specific interventions by national and county-level immunization stakeholders. Further studies on the relationship between attitude towards new vaccines and connection to vaccine hesitancy is necessary.

**Supplementary Information:**

The online version contains supplementary material available at 10.1186/s12889-023-15855-w.

## Introduction

Vaccination remains the most effective means of reducing the burden of infectious disease besides clean water and sanitation [1] and has been estimated to prevent between three to about six million vaccine-preventable deaths annually [2]. It is one of the most clinically and cost-effective public health innovations for promoting child health because of its direct health benefits and positive externalities [3, 4]. Vaccination has mitigated major epidemics of life-threatening diseases, eradicated many, besides being the surest prophylaxis against morbidity and mortality from vaccine-preventable diseases, and a significant contributor to national disease elimination and eradication efforts [5, 6].

However, despite the tremendous progress and being one of the most successful public health interventions yet, basic vaccination coverage remains below the 90% and 80% target at national and sub-national levels [7, 8]. An estimated 5.3 million child death from the 679 million under five years old (Under-5) in 2018, out of which over 700,000 are from vaccine-preventable infectious diseases, and 99% of them lived in low-and-middle-income countries (LIMC) [9]. In the Sub-Saharan Africa (SSA) region, basic vaccination coverage of diphtheria-tetanus-pertussis (DTP3) has stagnated at an average of 72% in the last decade, the lowest among World Health Organization (WHO) member regions, even lower than the global average of 86% [10, 11]. Before the Coronavirus (COVID-19) pandemic, about 20 million infants are either under or unvaccinated annually, most of whom are in SSA region, where deaths rate from vaccine-preventable-diseases (VPD) for children under-fives years old (Under-5) remains the highest in the world [12, 13].

**Fig. 1:**
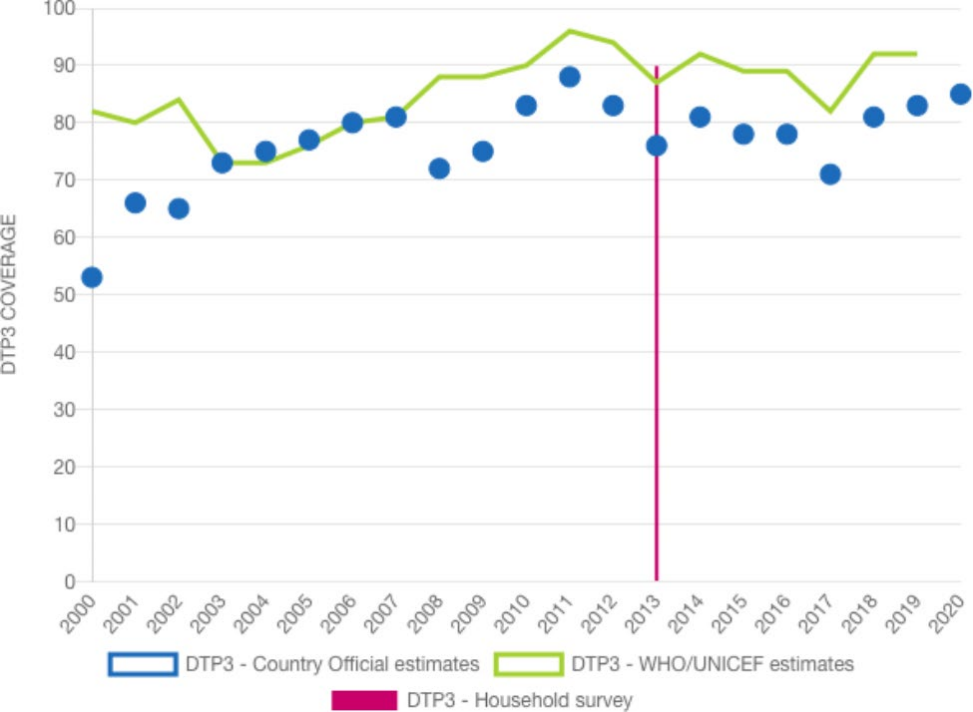
Kenya national basic childhood immunization trend [16]

In Kenya, basic childhood vaccination coverage was lower (82%) than the global average (86%) in 2020 [11, 14, 15]. The national DTP3 coverage has been inconsistent in the last decade as seen in Fig. 1 [16]. These accumulations of high unvaccinated children and the inconsistent coverage (due to vaccine hesitancy and medical inequality) for childhood and adolescent vaccination are major contributors to perennial childhood diseases outbreaks in Kenya and across the WHO AFRO region [4]. At the county levels, similar scenarios are observed too. While it has increased from 63% to 2000 to 83% in 2019, in quarter one of 2019, 21 Counties (44%) had 80% of DTP3 coverage but this dropped to 16 counties (34%) in quarter 2 [16]. There has been 68% stark differential in immunization coverage across the 47 counties in Kenya [17]. In 2017, only 6 of the 47 counties (13%) had basic vaccination (DPT3) coverage of at least 90%, while only 9% (four counties) had MMR 1 coverage of at least 95% – the lowest coverage reported in the country since 2011 [18, 19].

Similar trend (vaccine hesitancy) can also be noticed for Human papillomavirus (HPV) vaccination as well. HPV infection is the common cause of cervical cancer [20–22], with more than half of sexually active population contracting it during their lifespan [23]. While over 70% of all cervical cancer cases are attributed to HPV types 16 and 18, it is the second most prevalent type of cancer among women [20, 24]. In 2020, an estimated 604, 000 new cases and 342, 000 deaths occurred, and about 90% of these occurrences were in LIMC, predominantly in SSA [21, 25]. In 2018, 20 countries (except Bolivia) with the highest global burden of cervical cancer are in SSA and Kenya ranked 20th [26, 27].

Over 50% of women in Kenya who are diagnosed with HPV died from cervical cancer (i.e., nine death per day), with 14.3 million more women at risk of having cervical cancer [20, 28]. These deaths are a general reflection of the dire situation in LIMC, especially Kenya, where control strategy for cervical cancer remain inadequate and HPV vaccine provides the best protection. HPV vaccination was introduced in October 2019 for girls aged 10 years old in Kenya, in line with recommendations of the WHO’s Strategic Advisory Group on Immunization (SAGE), that HPV vaccine should be administered to young girls between the ages of 9–14 years before the onset of sexual activity [29].

Studies in Kenya have shown that vaccine acceptance is influenced by several factors including scheduling, knowledge gaps about immunization, behavioral components, including myths and misconceptions about vaccination [29, 30]. In 2018, according to the WHO/UNICEF Joint Reporting Form, three reasons reported to be driving vaccine hesitancy in Kenya are fear of adverse events from immunization (AEFI), religious belief and mythical or conspiratorial theories and misconception about vaccination [11]. However, these reasons are not grounded in empirical evidence. Similarly, empirical findings from a systematic review of 13 countries in the SSA (including Kenya) shows that low demand for HPV vaccine is associated with risk perception, concerns about its safety and effectiveness, inadequate knowledge, and awareness [31–33].

Therefore, the tremendous successes made over childhood immunization are still incomparable to the target, let alone the stagnant or reversal of some of these gains in the last decade owing to behavioral reasons and other vaccine-related controversies [34, 35]. The same applies to the newly introduced HPV vaccine. Kenya HPV vaccine uptake has been sub-optimal with only 33% of adolescent girls receiving the first dose in 2020 and half (16%) returning for the 2nd dose [29].

**Fig. 2:**
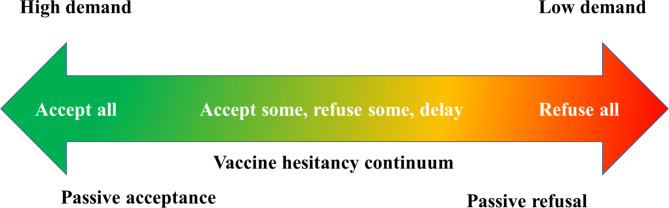
The continuum of vaccine hesitancy [36]

Studies have associated these vaccination stagnation, retrogression, or inconsistencies to vaccine hesitancy [6, 36–38]. Which was why WHO considered vaccine hesitancy as one of the top ten threat to global health [39]. Vaccine hesitancy is defined as the delay in acceptance or refusal of vaccines despite availability of its services [36]. As shown in Fig. 2, vaccine hesitancy is not an all-or-nothing situation, but a continuum of a process from acceptance to complete refusal. Vaccine hesitancy is also perceived to be driven largely by factors such as confidence (level of trust in vaccine or provider), complacency (do not perceive a need for vaccine or do not value the vaccine), and convenience (access) [40]. However, vaccine hesitancy is a complex and context-specific phenomenon, and depends on geographical locations, time, types of vaccines or even groups [41].

There is dearth of empirical data on the context-specific factors that drives low vaccine demand or vaccine hesitancy for routine childhood immunization and vaccination of adolescent girls against HPV in Kenya. The availability of data is crucial for the development of evidence-based targeted interventions to reduce vaccine hesitancy and improve both childhood and adolescent vaccination uptake. Therefore, this study aimed to identify the context-specific factors that influence vaccine hesitancy from the point of view of both the immunization program stakeholders/community members (supply-side) and caregivers (demand-side). It is important to assess both sides of the divide to enable comprehensive overview and design of potential multilayer intervention framework that are suitable to the Kenyan context.

## Methods

### Study design

The study design used qualitative research method such as Key Informant Interview (KII) and In-depth Interview (IDI). The methodological approaches are suitable because, the KII provided an in-depth understanding of contextual and social issues surrounding the phenomenon, while the IDI enhanced knowledge insights directly from the affected population. KII were conducted to obtain information from national and county-level representatives of the Expanded Program on Immunization (EPI) and key community stakeholders. IDI were used to obtained information from community members and caregivers (parents or legal guardian) of children 0–23 months and adolescent girls eligible for recommended routine childhood immunization and the HPV vaccine respectively. Participants were purposively selected based on relevance to vaccination decision-making in households, community, counties, and national levels.

### Study setting and population

**Fig. 3:**
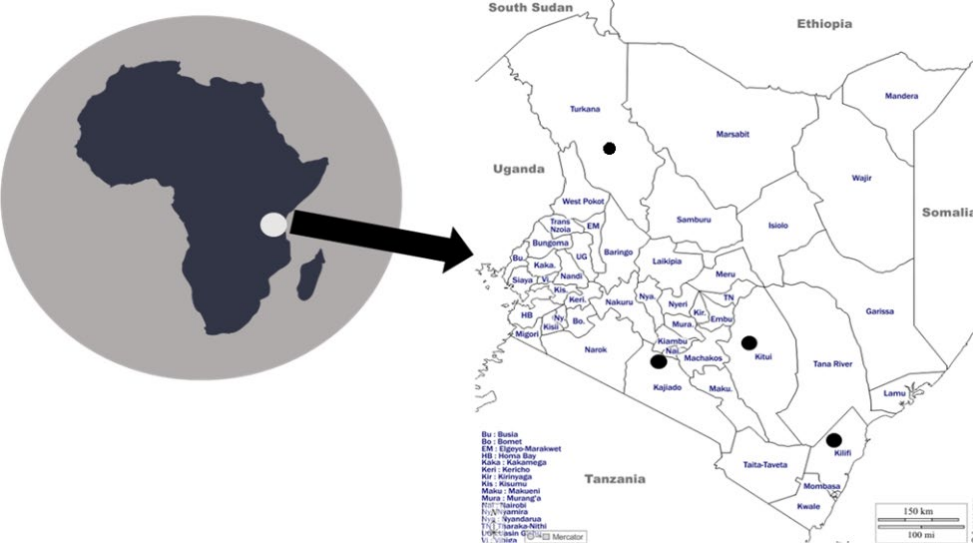
Map of Africa showing Kenya (white dot) and the participating counties (black dots)

This study was conducted in four counties: Kilifi, Turkana, Nairobi and Kitui (see Fig. 3). The selection of study sites was based on vaccination coverage such as high (Kilifi) and low (Turkana) counties, an urban county where the HPV vaccine was piloted (Nairobi) and a rural or sub-urban county where the same was done (Kitui). We purposively selected 41 key immunization stakeholders and caregivers for interview at both the national and county levels, because of the expert knowledge of the stakeholders and caregivers’ personal experience. The key actors comprised 25, while caregivers are 16 participants. At the central level, participants include the national EPI manager, EPI logistician, a NITAG member, UNICEF and WHO representatives. At each of the four county levels, the participant included the EPI manager, one HCW, one teacher each from a school where an HPV demo project was conducted, a religious leader (RL), a community leader (CL), two caregivers of children under five years old (Under-5) and two caregivers of adolescent girls aged 9–14 years old.

### Data collection

Data were collected from participants using an interview guide, designed both in English and Kiswahili, depending on the preference of the participants. The data collection was conducted between August 6 and September 6, 2020. The interviews lasted an average of 20 min and explored stakeholders and caregivers’ opinion regarding factors driving vaccine hesitancy in Kenya. The shortest and longest interviews lasted 17 and 25 min respectively. Interviews were recorded using an encrypted recorder and transcribed verbatim. Interviews done in Kiswahili were transcribed into English language.

### Themes explored

The themes explored during the interviews were broadly knowledge of immunization, knowledge of cervical cancer, attitudes about routine childhood immunization and HPV vaccine, and perceived barriers to vaccination. All caregivers were asked about childhood vaccination, but only the caregivers of adolescent girls were asked about cervical cancer and HPV vaccination. The English and Kiswahili languages interview guides are available as supplementary files.

### Data analysis

Inductive thematic content analysis was used to analysis the data. The main and sub-themes were identified and categorized after analyzing individual transcripts. Transcribed data were coded as follows: the KIIs were coded as KII001…KII022, while the IDIs were coded as IDI001…IDI016. We organized the factors driving vaccine hesitancy using the three dimensions of the SAGE vaccine hesitancy model, i.e., vaccine and vaccination-specific factors, individual and group factors, and contextual factors [36]. The vaccine and vaccination-specific issues are factors that relate to vaccines or vaccination, the individual and group influences relate to individual perceptions or their social environment; and the contextual influences relate to a range of factors such as historical, sociocultural, environmental, health system, institutional, economic, and political factors [36].

## Results

### ***Vaccine and vaccination factors*** driving vaccine hesitancy for routine childhood immunization

#### Fear of adverse event from immunization (AEFI)

Caretakers expressed fear about AEFI and that their children could end up being sick or worse after receiving the vaccine, especially when it was injection.

*“…when I took my first-born child for vaccination, his leg got swollen and I thought the injection was out-of-date. I was hesitant to return for the next visit”* - HCW (Kitui).

#### Shortage of vaccines

The participating managers reported that targets of vaccination coverage had been reached by some of the counties, although vaccine shortages due to the COVID-19 pandemic and other disruptions, such as HCWs industrial actions have an impact in other counties:

*“From January to March 2020, we had reached 25% of our target, so without the pandemic we could have probably reached 80% at the end of the year” –* EPI Logistician/HCW (Central/Nairobi). *“In August 2020, we had stock-outs of the pentavalent vaccine…I think there was a delay in the supply chains to the national level”* EPI Manager (Central).

#### Cost of transportation

Caregivers considered cost of commuting from their communities to healthcare facilities as a major constraint causing delay, missed appointments and even complete refusal of vaccination.

*“Some people are poor, and live far away, so getting to hospital is a challenge”* – HCW/Caregiver 1 (Kitui/Kilifi). *“Transport is so expensive, more than 500 shillings (about $4.5)...some use motorcycles from very rural places but it is a lot of money”* – Teacher/RL (Kilifi/Nairobi).

#### Constraints or competing priorities and long waiting hours

Caregiver complained of competing priorities influencing their vaccination decision-making. Some caregivers acknowledge the awareness that they were supposed to take their children for immunization but given a choice between abandoning work or important tasks for immunization, they prefer to forgo immunization. In addition, the long waiting time complicates vaccination decision of working caregivers, especially mothers.

*“Sometimes not everyone can bring their children for immunization because they have other errands to run”* – Caregivers 2/CL (Nairobi/Kitui;). *“When mothers come, and they wait many hours, they say: ‘probably next month, I may not come’…”* – HCW (Turkana).

#### General problem of stock-out of vaccines

Stock-out of vaccines seem a recurrent phenomenon in Kenya pre-pandemic and grew worse during the pandemic. *“This child who is 1.5 years old have not been vaccinated because whenever I could go to the clinic, we were told the vaccines were out of stock”* – HCWs (Kilifi/Kitui/ Turkana).

### Individual and group factors driving low demand or vaccine hesitancy for routine childhood immunization

#### Inadequate knowledge of vaccine-preventable-diseases

The caregivers lack the requisite knowledge of the advantages of vaccinating their children. Many would be happy for their children to receive vaccines if they properly understood what the vaccines do. The community HCW, and religious leaders said that vaccine hesitancy was often due to lack of correct information and awareness in the community. *“…we often hear something like, I don’t want my child to be vaccinated anyhow, is the baby sick*?” – CL/RL (Kilifi/Turkana).

#### Lack of incentives

Lack of incentive was cited, especially by the participants from the Turkana County.

*“Some mothers feel they need food at healthcare facilities and if it is not given, they do not see the need of going and staying long hours for the vaccine” –* HCW/EPI Manager/Caregiver 2 (Kilifi/Turkana). “W*hy are you giving these kids vaccines and you’re not giving them food before you give it to them?”* – Caregivers 1 (Turkana).

### Contextual factors vaccine hesitancy for routine childhood immunization

#### Fear of COVID-19 infection cum restriction of movement

Caregiver’s fear of COVID-19 infection resulted in cancellation of vaccination appointments. Also, communications about the COVID-19 pandemic response strategies (e.g., restriction on movement of people) were unclear and insensitive to healthcare services such as immunization; especially the directives from the Kenyan government to stay at home. In addition, many caregivers were turned back by HCWs due to fear of bringing COVID19 into the facilities. *“Our services were closed completely for almost two weeks and caregivers were told not to come…one day you turn a mother away and the next, you say come back, it was very confusing for them.”* – HCWs/EPI Manager (Nairobi/Kitui/Kilifi).

#### Religious influence

In Kenya there are certain religious sects, such as Roho and Legio Maria, that are against conventional medicine, including vaccination (Shikuku et al., 2019). Their views on vaccination have a negative influence on uptake among followers. *“There are people who say children survive by power of God and others who say their religion does not allow them to take children for vaccination, therefore they don’t believe in vaccines.”* – EPI Managers/ EPI Logistician (Central).

### ***Vaccine and vaccination factors driving vaccine hesitancy for*** HPV vaccine

#### Inadequate knowledge of HPV vaccine

Many caretakers knew about a vaccine that prevents cervical cancer, however, not all knew what the name of the vaccine was. For the few that knows about HPV vaccine, they thought it was a one-off vaccination.

*“The day I took my youngest child to the hospital, I was surprised my daughter aged nine got a vaccine they said was for girls only. I asked what vaccine it was, and they said it was a vaccine to prevent cervical cancer. I have not asked anyone else about that vaccine, but we were given a date to go back, so, I will ask about it then”* – Caregivers 2 (Turkana/Kilifi/Kitui). *“a number of parents do not know how many doses are given, and there are those who thought it is just a one-off campaign, so there is a gap in knowledge…”* – EPI Managers/CL (Kitui/Kilifi/Turkana).

#### Low literacy among caregivers

Low literacy level among caregivers contributed to low awareness about the importance of HPV vaccination:

*“…some caregivers are illiterate and don’t understand information about vaccine. I wish population could be empowered to understand the real importance of HPV vaccine”* – Teacher/EPI Manager (Kilifi/ Turkana).

### Individual and group factors driving low demand or vaccine hesitancy for HPV vaccine

#### Rumors and conspiracy theories associate with vaccination of girls

HPV vaccine is often misunderstood because of the beliefs that it is a vaccine meant to control population growth in Kenya. Some caregivers believe that the HPV vaccine makes girls infertile, hence the focus of the vaccine on girls. These rumors and conspiracy theories, which make caregivers reluctant to vaccinate, are predominant among some religious sects especially Kavonokya, Akorino, Legio, etc.

*“Some caregivers accused us of vaccinating their daughters with family planning vaccine…despite explanations many still declined”* – EPI Manager/HCWs (Kilifi/Turkana). *“I heard if HPV vaccine is given to girls, they will become infertile, they won’t give birth... that the government has a plan to reduce the population. That’s what I have heared within my community”* Caregivers (Kilifi/Turkana). *“The Kavonokya religious sect don’t go to the hospital… it is their religious tradition”. Some of the churches like Akorino, Legio do not allow children to receive injections. In their religion, they claim God will heal them of any diseases” –* EPI Managers (Nairobi/Kitui/Turkana).

#### The role of social media and caregivers in urban centres

The study revealed that caregivers of higher income brackets and residing in urban centers tends to seek information from the social media. This can lead to misinformation which results in vaccine hesitancy. Stakeholders emphasized that vaccination campaigns faced snob from high income groups, despite the expectation that this group are supposed to have better knowledge and pro-vax. *“We get hesitancy from the elite because, they believe that they know-how, we don’t need to go to them, they’re supposed to come to our facilities”* – EPI Manager (Nairobi).

### Contextual factors driving vaccine hesitancy for HPV vaccine

#### The COVID-19 pandemic and the response strategies adopted

The COVID-19 pandemic has had significant and negative impacts on general healthcare services including vaccination. Similarly, the response strategies adopted by the Kenyan government such as closure of schools and churches affected HPV vaccine uptake.

*“Because of COVID-19 pandemic, some parents say that their daughters will not come to the healthcare facilities, because they could contract the virus”* HCWs (Nairobi/Kitui/Kilifi). *“Now remember our venues where we normally do advocacy for this HPV vaccine, the schools and churches were closed, so, our hands were tied”* EPI Manager (Turkana).

#### Attitude of healthcare workers

Caregivers described the behavior of HCWs at the healthcare facility as turn-off. Caregiver described many of the HCWs to be disrespectful, they talk to them in such a disdain manner, and their attitudes in general are a barrier to willingness to return for subsequence vaccine doses. In many facilities, HCWs do not communicate well with caregivers when conveying vaccination information, especially vaccine stock-outs and possible dates to receive new stocks.

*“When caregivers go to healthcare facility, the HCWs mistreats them, they talk to them in a very rude manner. They don’t even explain why they are giving the vaccines” –* CL (KII020, Kilifi). *“Most caregivers would be willing to return if the HCWs attitude changes and are given accurate information on vaccine stocks, because sometimes the caregivers are not told anything other than, the vaccines are not available, so go away”* – CL/Caregivers (Nairobi/Kitui).

## Discussion

This study provides a unique window into the demand-side and supply-side of vaccination phenomenon in Kenya, in order to understand the influencing factors driving low-vaccination demand or enablers of vaccine hesitancy for both routine childhood immunization and HPV vaccination. Also, using two qualitative data approach (KIIs and IDIs), the study expanded the exploration of data beyond demand-side (caregivers) and supply-side (healthcare/EPI) program narratives, hence mashing opinions to produce a robust and clear understanding of the problems to aid intervention program. Involving different immunization and HPV vaccine stakeholders, including national and local key informants, and caregivers, are novelle and should be improved upon.

**Fig. 4:**
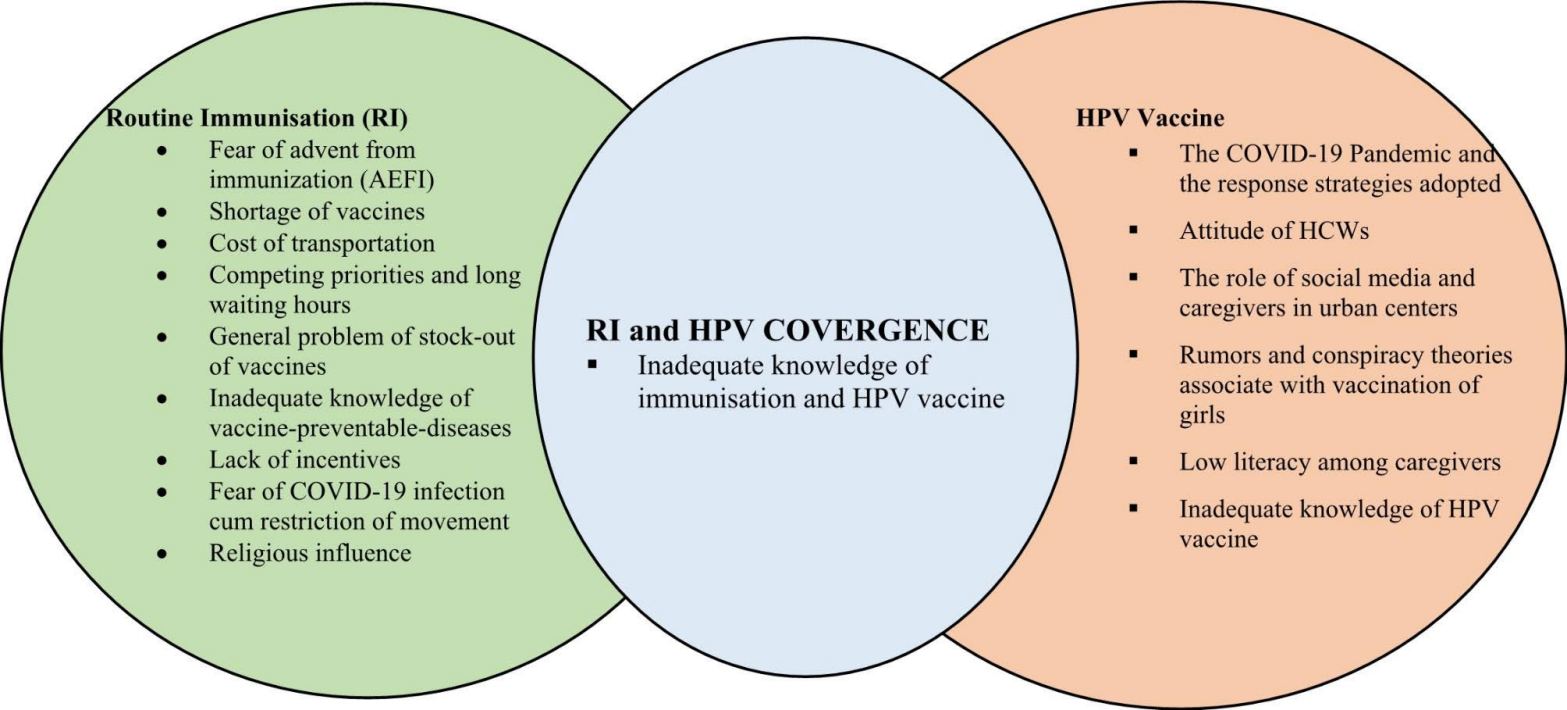
Overview of factors driving low vaccination demand or vaccine hesitancy in Kenya for RI (left), HPV vaccination (right), and both (middle). The data are from both KII and IDI. RI = Routine Immunization. HPV = Human Papillomavirus

Some of the reasons as shown in Fig. 4 for vaccine hesitancy identified were lack of knowledge about vaccines, fear of adverse events from immunization, belief in rumors about vaccines being used for inducing infertility, illiteracy among the community, inadequate knowledge of both RI and HPV vaccines. Additionally, hesitancy by caregivers in urban areas was reported to be associated with misinformation from social media. Also, over the years, vaccine successes that equates to absence of VPDs are slowly creating complacency, with the feeling of no obligation to vaccinate children or threats of VPDs are not there anymore.

Although there have been many studies focusing on vaccine hesitancy and acceptance, few have been conducted in LIMC that take into consideration their unique characteristics. Vaccine hesitancy varies over time and geographical regions and with different vaccines, therefore continuous monitoring and education should be provided to communities. Because in LMIC, participation is sometimes motivated by political, social and economic factors, e.g., provision of free medical care [42]. There are other limitations and enablers that need to be considered to improve vaccination uptake in Kenya or generally in LMICs, such as SMSs to mobile phones, which could be a cost-effective way to reach larger and hard-to-reach populations, to improve health outcomes and confidence in the healthcare system.

A study in Pakistan found that automated mobile phone-based personalized messages (SMS or automated call) improved childhood vaccination uptake at 6, 10, and 14 weeks of age compared with a usual care control group [43]. A Cochrane review concluded that vaccine uptake could be improved in LMIC by multifactorial interventions such as providing parents and other community leaders with immunization information and providing health education at healthcare facilities [3]. In addition, home visits, outreach vaccination programs, incentivizing caregivers, and integration of immunization and adolescent vaccine for girls into other services could improve vaccination uptake.

Communication from HCWs is crucial for education and reminding caregivers about what vaccines are available and what they do, particularly for new vaccine such as the HPV. This communication should be continuous, even when the vaccines are out of stock or when HCWs are on industrial actions. When the problems are resolved, i.e., the vaccines are in-stock or the industrial actions are called off, communication should be maintaind to inform the caregivers and remined them of the importance of resuming the vaccination schedules.

Healthcare workers should be involved in awareness campaigns for HPV vaccination as they are trusted by the communities. Involvement of other actors from the communities, such as religious and community leaders, school teachers, could improve awareness and strengthen acceptance for hesitant caregivers. In rural communities of large counties with distance between the communities and healthcare facilities, regular outreach immunization campaigns should be sustained to help to improve vaccination uptake. Extensive social mobilization and raising awareness are needed to demystify the myths and misconceptions that still exist about HPV vaccine. The same should also target concerns raised by various religious organizations.

The continued use of politicians, such as governors to provide political support and champion immunization programs, which has always had an impact on immunization activities, as well as increase confidence in immunization and reduce vaccine hesitancy should be encouraged. Both national and sub-national governments should increase public expenditure for immunization program and its communication activities, such as outreach educational programs and social media campaigns, that are important for increasing vaccination awareness and demand in urban centers. Also, social media must be a significant component of any vaccination uptake intervention strategies because it has capacity to counter misinformation and an effective communication tool for behavior change [44].

The main limitation of the study is the purposive sampling approach used. The approach is vulnerable to research bias; hence, researchers are confronted with subjective assumptions during participant’s selection process. However, selection of participants in this study was based on relevance to vaccination decision-making in households, community, counties, and national levels, hence the approach is suitable and appropriate.

## Conclusion

Low vaccination demand or vaccine hesitancy must be tackled through collective activities that increases awareness about importance of vaccination and must carry along all stakeholders. The restriction of movement as a response strategy to the COVID-19 pandemic, which limited accessibility to immunization services for most caregivers was a profound public health failure during this period. The consequence of such will have a long-term negative impact on general healthcare services, and immunization. Therefore, the lack of coordination with officials (Ministry of Health) involved in the healthcare system decision-making process in Kenya was a big lesson. The healthcare systems in the country were not consulted on how the restriction of movement or stay-at-home laws would impact on healthcare services, including immunization. It is essential for public health policy to be based on advice from health professionals and behavioral experts, especially during pandemics and health emergencies. These lessons must be carried into future pandemic preparedness and response.

Also, through this study, especially for HPV vaccine, it is apparently clear that adolescent vaccination programs must be linked to other healthcare promotion interventions that targets this age group, which hitherto have been isolated from many public health interventions in Kenya.

Grassroot and rural community sensitization on both routine childhood immunization and HPV vaccination should be key activities post COVID-19 pandemic. The use of mainstream and social media outreaches, and vaccine champions could help reduce vaccine hesitancy.

## Electronic supplementary material

Below is the link to the electronic supplementary material.

Supplementary Material 1

## Data Availability

The datasets used and/or analyzed during the current study are available from the corresponding author on reasonable request.
