# Supplementary material for: Exploring the factors contributing to low vaccination uptake for nationally recommended routine childhood and adolescent vaccines in Kenya
Source: BMC Public Health. 2023 May 19;23:912. doi: 10.1186/s12889-023-15855-w (PMC10197028; doi:10.1186/s12889-023-15855-w)
Supplement: Supplementary file 1 — Supplementary Material 1 [file 12889_2023_15855_MOESM1_ESM.pdf]

ANNEX 1A: KEY INFORMANT INTERVIEW (KII) GUIDE

This guide target EPI manager as well as staff (vaccine staff, communication officers) at the national and district level. Members of the committee responsible for immunization such as NITAG, etc. at the national level.

1. **Introduction**
   - **1.1** Introduction of the person to be interviewed and his role in vaccine implementation at his country level.
   - **1.2** Committee member (specify committee)?
   - **1.3** EPI staff (specify)
   - **1.4** Your age?
   - **1.5** Sex of the respondent?
2. **Knowledge of Immunisation and Current State of vaccine hesitancy**
   - 2.1 What do you think of immunization? Probe on uptake of vaccines, past and current situation?
   - 2.2 What is your understanding of vaccine hesitancy? Probe: Do you know, or have you heard of vaccine hesitancy? If so, can you tell us more about it?
   - 2.3 Do you speak in your committee, or between immunization keys actor's about vaccine hesitancy? If so, can you tell us what you discussed about vaccine hesitancy? and how often?
   - 2.4 Do you think that the vaccine hesitancy is an important topic for you and for your committee or organization? If yes, why?
   - 2.5 Do you think that the vaccine hesitancy has an impact on immunization? If yes, do you describe these impacts?
3. **Exposures to vaccine hesitancy (Experience and Existing Countermeasures)**
   - 4.1 Have you any experience or knowledge of vaccine hesitancy in Kenya? If so, when did they take place, or what were the populations and localities involved?
   - 4.2 From your awareness and experience of vaccine hesitancy, how did the actors act? Do you think they used the best approach, or the approach was limited on this specific case, why?
   - 4.3 Based on your experience or other experience, what do you think is the best approach to overcome reluctance to vaccination?
   - 4.4 Are there existing strategies at the country level in terms of prevention, communication and management of vaccine hesitancy that you know. If yes, what are these strategies?
4. **Knowledge and Perceived Attitude towards HPV vaccine or vaccine against cancer of the cervix**
   - 4.1 Do you know or have you heard of HPV vaccine against cervical cancer and HPV target? If yes, what do you think about HPV vaccine and who are the targets?
   - 4.2 Have you known or heard any discussion or information about HPV vaccine? If so, can you tell us about these discussions or information?
   - 4.3 Do you know or have heard about any hesitation of HPV vaccines? Where was that or among whom (groups)?
5. **Recommendations on vaccine hesitancy**
   - 5.1 What would be your recommendations to provide solutions to vaccine hesitancy?
   - 5.2 Can you give us two or three approaches that can be effective in addressing to vaccine hesitancy?
   - 5.3 How do you think it is necessary to implement the fight against vaccine hesitancy sustain?
   - 5.4 Who should we involve in fighting vaccine hesitancy?
   - 5.5 What will be the role of actors to fight against vaccine hesitancy and who should be actors /committees/institution?
6. Do you have any comments or questions from the discussion we have just had?

Thank you for your time and contribution

ANNEX 1B (TRANSLATION): MWONGOZO MUHIMU WA MAHOJIANO YA MTOA TAARIFA.

Habari muhimu inaweza kuwa meneja wa EPI na wafanyikazi (wafanyikazi wa chanjo, maafisa wa mawasiliano) katika ngazi ya kati na ya wilaya/ kaunti. Wajumbe wa kamati inayohusika na chanjo kama NITAG, nk katika ngazi ya nchi au maafisawanaohusika katika kutekeleza chanjo kama vile viongozi wa jamii, viongozi wa dini na walimu wanaohusika katika chanjo ya HPV.

1. **Utangulizi**
   1.1 Utangulizi wa mtu kuhojiwa na jukumu lake katika utekelezaji wa chanjo katika ngazi ya nchi yake.
   1.2 Mjumbe wa Kamati (taja kamati)?
   1.3 Wafanyikazi wa EPI (taja)
   1.4 Umri wako?
   1.5 Jinsia ya mhojiwa?

2. **Ujuzi wa chanjo na jambo la sasa la kusita / ukataaji wa chanjo**
   2.1 Je, ni nini maoni yako kuhusu huduma za chanjo? Uliza; kuhusu utumiaji wa chanjo, hali ya zamani na ya sasa?
   2.2 Ni nini ufahamu wako juu ya kusita/ kukataa kwa chanjo? Uliza: Je, unajua, au umesikia juu ya kusita/ kukataa kwa chanjo? Ikiwa ni hivyo, unaweza kutuambia zaidi juu yake?
   2.3 Je, huwa unazungumza katika kamati yako au kati ya washirika wakuu wa chanjo kuhusu kusita / kukataa kwa chanjo? Ikiwa ni hivyo, unaweza kutuambia ulijadili nini juu ya kusita / kukataa kwa chanjo? Na ni mara ngapi?
   2.4 Je, unafikiria kwamba kusita / kukataa kwa chanjo ni mada muhimu kwako na kwa kamati yako au shirika lako? Ikiwa ndio, kwanini?
   2.5 Je, unafikiria kwamba kusita /kukataa kwa chanjo ina athari kwa chanjo? Ikiwa ndio, unaweza nielezea athari hizi?

3. **Maelezo juu ya kusita/ kukataa kwa chanjo (Uzoefu na mipangilio zilizopo)**
   3.1 Je, una uzoefu wowote au ufahamu wa kusita/ kukataa kwa chanjo in Kenya? Ikiwa ni hivyo, zilifanyika lini, au ni idadi gani ya watu na maeneo yapi yalihusika?
   3.2 Kutoka kwa ufahamu wako na uzoefu wa kusita / kukataa kwa chanjo, watendaji chanjo walifanya nini? Je, unafikiri walitumia njia bora, au mbinu ilikuwa inatosheleza kesi hii maalum pekee, kwa nini?
   3.3 Kulingana na uzoefu wako au uzoefu mwingine, unafikiri ni njia bora ya kushinda kusita kwa chanjo?
   3.4 Je, kuna mikakati iliyopo katika ngazi ya nchi kwa suala la kuzuia, mawasiliano na usimamizi wa kusita/kukataa kwa chanjo ambayo unaijua. Ikiwa ndio, mikakati hizi ni zipi?

4. **Ujuzi na mtazamo uliopatikana kuhusu chanjo ya HPV**
   4.1 Je, unajua au umesikia kuhusu chanjo ya HPV na malengo ya HPV? Ikiwa ndio, ni nini maoni yako kuhusu chanjo ya HPV na inalenga akina nani?
   4.2 Je, umejua au kusikia mazungumzo yoyote au habari juu ya chanjo ya HPV? Ikiwa ni hivyo, unaweza kutuambia juu ya majadiliano haya au habari hiyo?
   4.3 Je, unajua au umesikia juu ya kusitishwa / kukataa kwa chanjo ya HPV? Hiyo ilikuwa wapi au kati ya nani (vikundi)?

5. **Mapendekezo juu ya kusita / kukataa kwa chanjo**

5.1 Je, ni nini mapendekezo yako ya kutoa suluhisho la kusita / kukataa kwa chanjo?
5.2 Je, unaweza kutupa njia mbili au tatu ambazo zinaweza kufaa katika kushughulikia jambo la kusita / kukataa kwa chanjo?
5.3 Je, unafikiria ni nini muhimu inaweza tekelezwa kwa mapambano dhidi ya kusita / kukataa kwa chanjo?
5.4 Ni nani tunapaswa kumshirikisha kupambana na kusita / kukataa kwa chanjo?
5.5 Je, ni jukumu gani watendaji wanayo kwa kupigania dhidi ya kusita / kukataa wa chanjo na nani wanapaswa kuwa watendaji / kamati / taasisi?

6. Je, una maoni yoyote au maswali kutoka kwa majadiliano ambayo tumekuwa nayo hivi karibuni?

Asante kwa wakati wako na mchango

ANNEX 2A: IN-DEPTH INTERVIEW (IDI) GUIDE - COMMUNITY MEMBERS

This guide targets community members involved in implementing immunization such as community leaders, community health volunteers, religious leaders and teachers involved in vaccination

1. Knowledge and perception of vaccination programmes

1.1 What are the common diseases that affect children in your community?
1.2 Which of them do you think can be prevented with vaccines? Probe why
1.3 Are there vaccines that target young people especially adolescent girls? What diseases do you think they can prevent?
For community and religious leaders ask:
1.4 Are you involved in the monitoring of child vaccination in your community? What is your role? *Probe for EPI vaccines*
1.5 Tell me about HPV vaccination and your involvement with the HPV vaccination exercise in your community
1.6 Are there any obstacles you encounter in monitoring child vaccination? *Probe on vaccine hesitancy at the community.*
1.7 What do you feel about the immunization services that is provided in your community? *Probe with accessibility: geographic, financial, availability relative to the vaccination calendar*
For teachers ask:
1.8 *What do you know about HPV? And what was your role in the HPV vaccination programme?*
1.9 *What would you say worked well in the HPV vaccination programme? Probe for training, availability of the vaccines, coordination with health workers)*
1.10 Were there any challenges encountered while supporting HPV vaccination? _Probe for timing for vaccination

2. Vaccine importance

2.1 Do you think immunization is an important topic in your community? If yes, why? *Probe issues around importance in the community.*
For community & religious leaders ask:
2.2 Do you often talk / discuss immunization of children and girls in your community? *If yes, probe discussion at the community level or between parents, girls and teachers.*
2.3 Who decides on vaccinating child in your community? *Probe with permission and request for counselling and who is involve (father, mother, grandparents, community leader, religious leader)*
2.4 In your community or at school, are there occasions when you talk about immunization? If yes, which ones? *Probe with opportunities to talk about community immunization.*

For teachers ask:

2.5 Are there discussions on vaccines in school? Probe how often these talks occur, what is covered?

2.6 *Who makes the decision on vaccination of children at the school? Probe for parents, teachers, guardians*

2.7 How are discussions on vaccines conducted in your school? *Probe on who facilitates information, the government, NGOs, who is involved girls, boys, teachers*

3. Hesitancy towards routine infant vaccines

3.1 What do you feel about vaccinating babies for common childhood diseases?
3.2 Do you think all babies in your community should be vaccinated? Probe reasons
3.3 Will you vaccinate your babies for common childhood diseases? Probe reasons

3.4 What do you think are the reason why some parents refuse or accept vaccination in this community? Probe reasons

4. Hesitancy towards HPV vaccine

4.1 What do you feel about vaccinating adolescent girls aged 10 and above for HPV? If so, *probe for details.*

4.2 Would you be willing to vaccinate your own adolescent girls against HPV? If Yes, *Probe on the confidence in the vaccine.* If not, why? *Probe for the concern*

4.3 Why do you think parents accept or refuse HPV vaccination? Probe for community sensitization, religious and other sociocultural barriers.

5. Improving vaccination programmes

5.1 What are your suggestions for improving overall childhood immunization in your community?

5.2 What are your suggestions for improving HPV vaccination in your community?

Thank the participant and end the interview

ANNEX 2B (TRANSLATION): MWONGOZO WA MAHOJIANO YA KINA - WANAJUMUIYA

Mwongozo huu unawalenga washiriki wa jamii waliohusika katika kutekeleza chanjo kama vile viongozi wa jamii, wafanyikazi wa afya wa nyanjani, viongozi wa dini na walimu wanaohusika katika chanjo.

1. **Ujuzi na mtazamo wa mipango ya chanjo**
   1.1 Je! Ni magonjwa gani ya kawaida ambayo yanaathiri watoto katika jamii yako?
   1.2 Ni ipi kati yao unadhani inaweza kuzuiwa na chanjo? Chunguza kwanini
   1.3 Je, kuna chanjo ambazo zinalenga vijana haswa wasichana wadogo? Je! Unafikiria ni magonjwa gani ambayo yanaweza kuzuia?

Kwa viongozi wa jamii na kidini uliza:

1.4 Je! Unahusika katika ufuatiliaji wa chanjo ya watoto katika jamii yako? Jukumu lako ni nini? Chunguza chanjo za EPI
1.5 Nieleze kuhusu chanjo ya HPV na kuhusika kwako na zoezi la chanjo ya HPV katika jamii yako
1.6 Je! Kuna vizuizi vipi ambavyo unakumbana navyo katika kuangalia chanjo ya watoto? Chunguza usumbufu wa chanjo kwenye jamii.
1.7 Unasikia nini juu ya huduma za chanjo ambazo hutolewa katika jamii yako? Chunguza ufikiaji: kijiografia, kifedha, upatikanaji wa jamaa na kalenda ya chanjo

Kwa waalimu uliza:

1.8 Unafahamu nini kuhusu HPV? Je! Jukumu lako katika mpango wa chanjo ya HPV ilikuwa nini?
1.9 *Je! Ungesema ni nini ilitendeka vizuri katika mpango wa chanjo ya HPV? Chunguza kuhusu mafunzo, upatikanaji wa chanjo, uratibu na wafanyikazi wa afya)*
1.10 *Je! Kulikuwa na changamoto yoyote ulikumbana nayo wakati wa kusaidia kwa chanjo ya HPV? Chunguza muda wa chanjo*

2. **Umuhimu wa chanjo**

   2.1 Je! Unafikiri chanjo ni mada muhimu katika jamii yako? Ikiwa ndio, kwanini? Chunguza kuhusu maswala inazingatia umuhimu wake katika jamii.

Kwa viongozi wa jamii na wa kidini uliza:

2.2 Je! Unazungumza mara nyingi / kujadili chanjo ya watoto na wasichana katika jamii yako? Ikiwa ndio, chunguza majadiliano katika ngazi ya jamii au kati ya wazazi, wasichana na waalimu.
2.3 Ni nani anayeamua juu ya chanjo ya mtoto katika jamii yako? Chunguza kwa ruhusa na ombi la ushauri na ni nani anayehusika (baba, mama, babu, babu ya kiongozi wa jamii, kiongozi wa dini)
2.4 Katika jamii yako au shuleni, kuna wakati unapozungumzia juu ya chanjo? Ikiwa ndio, ni ipi? Chunguza na fursa za kuzungumza juu ya chanjo ya jamii.

Kwa waalimu uliza:

2.5 Je! Kuna majadiliano juu ya chanjo shuleni? Chunguza mara ngapi mazungumzo haya hufanyika, ni nini huangaziwa?

2.6 Nani hufanya uamuzi kuhusu kuchanja watoto shuleni? Chunguza ikiwa ni wazazi, waalimu, walezi
2.7 Mazungumzo juu ya chanjo hufanywaje katika shule yako? Chunguza ni nani anayewezesha habari, serikali, mashirika yasiyo ya serikali, ambazo zinahusika na wasichana, wavulana, waalimu

3. **Kusita kwa chanjo za watoto wachanga**
   3.1 Unajihisi vipi kuhusu chanjo ya watoto kwa magonjwa ya kawaida ya utoto?
   3.2 Je! Unafikiri watoto wote kwenye jamii yako wanapaswa kupewa chanjo? Chunguza sababu
   3.3 Je! Utachanja watoto wako kwa magonjwa ya kawaida ya utotoni? Chunguza sababu
   3.4 Je! Unafikiria ni kwanini wazazi wengine walikataa au kukubali chanjo katika jamii hii? Chunguza sababu

4. **Kusita kwa chanjo ya HPV**
   4.1 Je! Unajihisi vipi kuhusu chanjo ya wasichana wa umri wa miaka 10 na zaidi ya HPV? Ikiwa ni hivyo, uchunguzi kwa maelezo.
   4.2 Je! Ungekubali kuchanja wasichana wako dhidi ya HPV? Ikiwa Ndio, chunguza juu ya ujasiri katika chanjo. Ikiwa sivyo, kwa nini? Chunguza ikiwa wasiwasi
   4.3 Je! Unafikiria ni kwa nini wazazi wanakubali au wanakataa chanjo ya HPV? Chunguza uhamasishaji wa jamii, vizuizi vingine vya kidini na kijamii.

5. **Kuboresha mipango ya chanjo**

   5.1 Je! Ni maoni gani yako ya kuboresha chanjo ya utotoni kwa watoto katika jamii yako?

   5.2 Je! Ni maoni gani yako ya kuboresha chanjo ya HPV katika jamii yako?

Mshukuru mshiriki na umalizie mahojiano

ANNEX 3A: IN-DEPTH INTERVIEW (IDI) GUIDE – CAREGIVERS (PARENTS/LEGAL GUARDIAN)

This guide target caregivers of infants eligible for routine immunisation and adolescent eligible for HPV vaccination at county level. Caregivers will be either mother, father or legal guardian.

1. **Introduction**

1.1 I will start by asking you to introduce yourself. Please tell me How old are you? What is the highest level of education that you attained, how many children do you have? What do you do for a living?

2. **Knowledge, Perception and Attitude about Vaccination**

2.1 What are the common health problems in your community? Probe: common health problems affecting children.

2.2 Tell me what you understand by immunization. Probe: what do you feel about getting an immunization and why? what vaccines are children and adolescents supposed to receive? Probe: how have you ensured that your child receives all the required vaccination?

3. **Information Sources and Access-related Constraints about Vaccination**

3.1 How do you get information about vaccines and vaccination? Check for hospital, church, social media, community health volunteer etc

3.2 How is immunization provided in your community? Probe: where do you receive immunization services from (check for hospital, home, church).

3.3 How do you think immunizations should be provided? Probe with accessibility: geographic, financial, availability relative to the vaccination calendar.

3.4 In your experience, how would you describe your punctuality to immunization appointments? Have you missed one/a few before? Why?

4. **Vaccine importance, Vaccination Decision-Making and Ecological Influence**

4.1 Do you think immunization is an important topic in your family? If yes, why? Probe: Who makes the decisions about immunization within the family.

4.2 How often do you discuss about immunization of children and girls in your family? probe who starts the conversation

4.3 **For caregivers with girls 10 years of age – eligible for HPV vaccination:** Who decides on vaccinating your girl in your family? Probe with permission and request for counselling and who is involve (father, mother, grandparents, teachers for HPV)

4.4 In your community, are there occasions when you talk about immunization? If yes, which ones? Probe with opportunities to talk about community immunization (community gatherings, women, men and youth meetings).

5. **HPV vaccine and cervical cancer**

5.1 Let’s go to cancers that affect women in this community. Have you ever heard about cervical cancer? Probe: from where/how? How serious of a problem do you think it is? What do you think should be done about it? Probe:

5.2 Do you know any vaccine that can be used to prevent cervical cancer? Probe: Where do you think such vaccines can be obtained and for who?

5.3 Do you know about a vaccine that can prevent cervical vaccine that is available in the country? What is your opinion about vaccinating young girls with HPV vaccine to prevent cervical cancer? Probe: What do you think are the reason why people are accepting it, what do you think are the reasons why people are refusing it?

5.4 **For caregivers whose daughters have received HPV vaccination:** How do you feel about the HPV vaccine that was administered to your daughter? Probe: How did you make the decision to vaccinate her? Where was it administered? What are the reasons why you allowed it for your daughter? Before receiving, did you initially doubted, refused or hesitated to vaccinate your daughter for HPV? Why? Why do you think some people accept it? Why do you think some people refuse it?

6. Vaccine hesitancy with regards to routine immunization

6.1 Have you ever doubted, refused or hesitated to vaccinate your children or self? Why? Probe for specifying reason (fear of side effects, fear of sterility, etc).

6.2 How do you feel about safety and effectiveness of vaccines generally? Probe, do you trust vaccines to protect?

6.3 In your community, do you know any parents who has been reluctant or refused to vaccinate their children? If so, can you share specific cases and what were the reasons for their hesitancy?

6.4 In relation to all these problems, what are your suggestions for improving vaccine coverage or vaccine acceptance in your household and in your community? Probe on local solution proposals.

6.5 Are there traditional or cultural practices that may make immunising your child difficult? Probe: How does your religion perceive vaccination? Population control.

6.6 In your experience, what makes getting vaccinated easy, what makes it difficult? Probe for childhood vaccines and HPV. If easy what makes it easy? If difficult how did you overcome them?

Do you have any comments, questions on the topic that we have just discussed?
Thank you for your time and contribution

ANNEX 3B (TRANSLATION): MWONGOZO WA MAHOJIANO YA MOJA KWA MOJA KWA WAZAZI NA WATUNZAJI

Haya mahojiano yanawalenga walezi wa watoto wanaostahili chanjo ya kawaida na kijana anayestahili chanjo ya HPV katika sehumu hii ya kaunti. Walezi watakuwa mama, baba au mlezi katika kaunti.

1. **Utangulizi**
   1.1 Nitaanza kwa kukuuliza ujitambulishe. Tafadhali niambie una umri gani? Ni kiwango gani cha juu kabisa cha elimu ambacho umepata, una watoto wangapi? Unafanya kazi gani?

2. **Maarifa, Mtizamo na Tabia juu ya Chanjo**
   2.1 Ni shida gani za kawaida za kiafya ambazo ziko katika jamii yako? Fuatilia: shida za kiafya za kawaida zinazoathiri watoto.
   2.2 Niambie unaelewa nini kuhusu chanjo. Fuatilia: unasikia nini juu ya kupata chanjo na kwa nini? Ni chanjo gani watoto na vijana wanapaswa kupokea? Fuatilia: umehakikisha vipi kuwa mtoto wako anapata chanjo yote inayotakiwa?

3. **Vyanzo vya Habari na Vizuizi vinavyohusiana kwa upataji wa Chanjo**
   3.1 Je! Unapataje habari kuhusu chanjo na kuchanjwa? Angalia kama ni hospitali, kanisa, mitandao za kijamii, madaktari wa nyanjani nk
   3.2 Ni vipi chanjo hupeanwa katika jamii yako? Fuatilia: unapokea wapi huduma za chanjo kutoka (angalia hospitali, nyumbani, kanisa).
   3.3 Je! Unafikiri chanjo inapaswa kupeanwa vipi? Fuatilia ufikiaji: kijiografia, kifedha, upatikanaji wa jamaa na kalenda ya chanjo.
   3.4 Katika uzoefu wako, unaweza kuelezeaje uwekaji wako wa wakati wa miadi ya chanjo? Je! Umekosa moja / chache kabla ya? Kwa nini?

4. **Umuhimu wa chanjo, Uamuzi wa Chanjo na Ushawishi wa Ekolojia**
   4.1 Je! Unafikiri chanjo ni mada muhimu katika familia yako? Ikiwa ndio, kwanini? Fuatilia: Nani hufanya maamuzi juu ya chanjo ndani ya familia.
   4.2 Je! Unajadili mara ngapi kuhusu chanjo ya watoto na wasichana katika familia yako? Fuatilia ambaye anaanza mazungumzo
   4.3 **Kwa walezi na wasichana wenye umri wa miaka 10 - wanaostahiki chanjo ya HPV**: Ni nani anauamuzi kwa kuchanja msichana wako katika familia yako? Fuatilia kwa idhini na ombi la ushauri nasaha na ni nani anayehusika (baba, mama, babu, babu kwa mwalimu wa HPV)
   4.4 Kwenye jamii yako, je! Kuna matukio wakati munazungumzia juu ya chanjo? Ikiwa ndio, ni ipi? Fuatilia na fursa za kuzungumza juu ya chanjo ya jamii (mikusanyiko ya jamii, wanawake, mikutano ya wanaume na vijana).

5. **Chanjo ya HPV na saratani ya kizazi**
   5.1 Twende kwa saratani zinazoathiri wanawake katika jamii hii. Je! Umewahi kusikia kuhusu saratani ya mlango wa kizazi? Fuatilia: kutoka wapi / vipi? Je! Unafikiri ni tatizo kubwa kiasi gani? Je! Unafikiria nini ifanyike kuhusu hilo?
   5.2 Je! Unajua chanjo yoyote ambayo inaweza kutumika kuzuia saratani ya kizazi? Fuatilia: Je! Unafikiria chanjo hizo zinaweza kupatikana na kwa nani?
   5.3 Je! Unajua juu ya chanjo ambayo inaweza kuzuia chanjo ya kizazi ambayo inapatikana nchini? Je! Maoni yako ni nini juu ya chanjo ya wasichana wadogo na chanjo ya HPV kuzuia saratani ya kizazi? Fuatilia: Je! Unafikiri ni sababu gani watu wanaikubali, unafikiri ni sababu gani watu wanazikataa?
   5.4 **Kwa walezi ambao binti zao wamepokea chanjo ya HPV**: Je! Unahisi vipi kuhusu chanjo ya HPV ambayo ilipewa binti yako? Fuatilia: Je! Ulifanyaje uamuzi kwa kuchanjwa kwake? Ilipeanwa wapi? Je! Ni kwa nini uliiruhusu binti yako?

Kabla ya kupokea, je! Uliwahi kutilia shaka, kukataa au kusita kumchanja binti yako kwa HPV? Kwa nini? Je! Unafikiria ni kwanini watu wengine wanakubali? Je! Unafikiria ni kwanini watu wengine wanakataa?

6. **Kusisitiza chanjo kuhusu chanjo ya kawaida**

6.1 Je! Umewahi kutilia shaka, kukataa au kusita kuchanja watoto wako au wewe mwenyewe kuchanjwa? Kwa nini? Fuatilia kwa kubainisha sababu (woga wa athari, woga wa kuzaa n.k).
6.2 Je! Unahisije usalama na ufanisi wa chanjo kwa ujumla? Probe, unaamini chanjo kwa kulinda?
6.3 Kwenye jamii yako, je! Unajua wazazi wowote ambao wamesita au kukataa chanjo ya watoto wao? Ikiwa ni hivyo, unaweza kushiriki kesi maalum na ni nini sababu za kusita kwao?
6.4 Kuhusiana na shida hizi zote, ni nini maoni yako ya kuboresha chanjo au kukubalika kwa chanjo katika nyumba yako na katika jamii yako? Fuatilia maoni ya suluhisho la eneo lake.
6.5 Je! Kuna mazoea ya jadi au kitamaduni ambayo yanaweza kumfanya kumchanja mtoto wako kuwa ngumu? Probe: Je! Dini lako linaonaje chanjo? Udhibiti wa idadi ya watu
6.6 Katika uzoefu wako, ni nini hufanya chanjo iwe rahisi, ni nini hufanya iwe ngumu? Fuatilia kwa chanjo za utotoni na HPV. Ikiwa ni rahisi kufanya nini iwe rahisi? Ikiwa ni ngumu ulishindaje?

Je! Una maoni yoyote, maswali juu ya mada ambayo tumejadili hivi karibuni?
Asante kwa wakati wako na mchango wako.
